# Toxicological Characterization of Ten Medicinal Plants of the Beninese Flora Used in the Traditional Treatment of Diarrheal Diseases

**DOI:** 10.1155/2021/6676904

**Published:** 2021-04-28

**Authors:** Tamègnon Victorien Dougnon, Edna Hounsa, Eric Agbodjento, Hornel Koudokpon, Boris Legba, Kafayath Fabiyi, Anny Afaton, Kévin Sintondji, Benoît Akpode, Jean Robert Klotoé, Fidèle Tchobo, Honoré Bankole, Tossou Jacques Dougnon

**Affiliations:** ^1^Research Unit in Applied Microbiology and Pharmacology of Natural Substances, Laboratory of Research in Applied Biology, University of Abomey-Calavi, 01 P.O. Box 2009, Cotonou, Benin; ^2^Training and Research Laboratory in Applied Chemistry, Polytechnic School of Abomey-Calavi, University of Abomey-Calavi, Abomey-Calavi, Benin

## Abstract

The use of medicinal plants in traditional medicine is a common practice in developing countries. However, this unregulated or poorly rational use may present a dose-dependent risk of toxicity to humans. This study aimed to explore the phytochemical and toxicological characteristics of ten (10) plant species used in the traditional treatment of infectious diarrhea in Benin. The acute toxicity of aqueous and hydroethanolic extracts of *Khaya senegalensis*, *Daniellia oliveri*, *Rauvolfia vomitoria, Vernonia amygdalina*, *Manihot esculenta*, *Ocimum gratissimum*, *Senna italica*, *Diospyros mespiliformis*, *Pterocarpus erinaceus,* and *Anacardium occidentale* was evaluated following the OECD 423 protocol at a single dose of 2000 mg/kg. This safety test was complemented by a larval cytotoxicity test. Hematological and biochemical examinations, as well as a histological study of the liver and kidneys, were performed. Larval cytotoxicity was assessed by the sensitivity of *Artemia salina* larvae to different concentrations of the plant extracts studied. Testing for chemical compounds was performed on the basis of differential staining and precipitation reactions. The mean lethal concentration (LC_50_) was determined by the probit method. The qualitative phytochemical screening of the plants studied revealed the presence of catechic tannins, gallic tannins, flavonoids, anthocyanins and sterol-terpenes, alkaloids, saponosides, and reducing compounds. This composition varied according to the plants studied. Acute toxicity data indicated that there was no mortality and no structural and functional alterations of the liver and kidneys of treated animals. Larval cytotoxicity data suggest that the plants studied are not cytotoxic (LC_50_ _≥_ 0.1 mg/mL). These observations reflect the safety of these plants and justify their use in traditional medicine in the treatment of many diseases including diarrheal diseases.

## 1. Introduction

Traditional medicine based on the use of medicinal plants is the first reflex of more than 80% of the world's population for primary health care [1–3]. In developing countries such as Benin, this widely accepted trend is an ancestral medical practice that is transmitted from generation to generation [4, 5]. The real effectiveness, accessibility, and low cost of medicinal recipes are the main reasons for the perpetuation of this endogenous practice [6, 7]. The plants used in the African pharmacopeia are directed against several diseases, particularly infectious ones. The prevalence of these infectious diseases is essentially linked to factors such as precarious water quality, unsanitary conditions, and poor food hygiene that are common to most developing countries [8, 9]. Diarrheal diseases are among the most deadly infectious diseases, particularly among children. In fact, every year, it is estimated that there are 2.5 billion cases of diarrhea in children under the age of five [10]. Ugboko et al. [11] reported that childhood diarrhea affecting children five years old and below accounts for approximately 63% of the global diarrhea burden. 15% of these children die [12].

Benin is one of the developing countries in which diarrhea diseases are one of the main causes of morbidity [13, 14]. Indeed, they have a direct impact on the costs associated with seeking health care, including several factors such as consultation, medication, and, in some cases, hospitalization which represents a burden on household expenditures [15]. The pathogens of diarrheal diseases are mainly bacteria [16]. Over the years, medical therapy based on the use of conventional antibiotics has shown not only its effectiveness but also its limitations. Indeed, most bacteria responsible for diarrheal episodes develop resistance to the antibiotics used in therapy.

The phenomenon of multiresistance is today a real public health problem for the effective management of infectious diseases. This problem of bacterial resistance combined with the cost and sometimes difficult access to antibiotics by populations reinforces the use of herbal remedies by these populations. World Health Organization has supported this idea by launching a diarrheal disease control program based on traditional medicine practices and prevention approaches [17]. This can have valuable benefits in reducing child mortality rates in developing countries. In several African pharmacopeias, several ethnobotanical studies have provided information on the richness of African flora in the traditional management of diarrheal diseases [18–20]. Benin, a West African country, has a great floristic diversity to which is added a centuries-old traditional use of plants with unsuspected therapeutic virtues. Akoegninou et al. [21] estimated the ethnobotanical potential of Benin's flora at 2807 plant species. *Khaya senegalensis, Daniellia oliveri, Rauvolfia vomitoria, Vernonia amygdalina, Manihot esculenta, Ocimum gratissimum, Senna italica, Diospyros mespiliformis, Pterocarpus erinaceus,* and *Anacardium occidentale* are some of the medicinal plants of the Beninese flora used in the traditional treatment of diarrheal diseases. These plants are also used in the traditional treatment of other diseases. For example, *Khaya senegalensis, Ocimum gratissimum, Daniellia oliveri*, and *Manihot esculenta* are used in the traditional treatment of metabolic diseases such as diabetes and blood disorders such as anemia [22–25]. The therapeutic virtues of these medicinal plants are attributed to secondary metabolites such as saponosides, flavonoids, coumarins, tannins, alkaloids, mucilages, volatile compounds, sterols, and terpenes identified in different parts of these plants [26]. However, despite the widespread use of traditional herbal remedies in the treatment of diarrheal diseases, very few scientific studies exist at this stage on the safety of antidiarrheal plants. It is, therefore, important, if not essential, to explore the toxicological characteristics of the main plants used by local communities in several African pharmacopeias in order to secure their use in traditional medicine. This study was initiated to produce recent data on the toxicological characteristics of selected plants as used in traditional medicine. It aimed to evaluate the larval cytotoxicity and acute toxicity of aqueous and hydroethanolic extracts of the selected plants on Wistar albino rats to predict their safety in the human species.

Rats are recommended lower-level animals for toxicity studies to extrapolate to human biology according to Organization for Economic Cooperation and Development (OECD) safety study guidelines [27, 28]. The finding of the study could also help to guide the optimization and validation of the traditional use of these antidiarrheal plants.

## 2. Materials and Methods

### 2.1. Plant Material

The plant material used in this study consists of plant organs from 10 medicinal plants used in the traditional treatment of diarrheal diseases in Benin (Table 1). These organs were harvested in various regions of Benin according to the indications of practitioners of traditional medicine. The plant samples once collected were authenticated at the National Herbarium of Benin using the analytical flora of Akoegninou et al. [21]. The botanical nomenclature of The PlantList available on the website http://www.Theplantlist.com has been used to confirm this identification.

### 2.2. Animal Material

The animal material consisted of eggs of *Artemia salina* and albino Wistar rats. Eggs of *Artemia salina* (ARTEMIO JBL D-67141 Gmbh Neuhofem) were used for the larval cytotoxicity test of selected medicinal plants. Three-month-old Wistar albino rats weighing between 130 and 180 g, all nonpregnant and female, from the pet shop of the Institute of Applied Biomedical Sciences (ISBA) of the University of Abomey-Calavi were used for acute toxicity testing. Upon receipt, the rats were randomly assigned to groups of 3 in standard cages for a 2-week acclimatization period in the Research Unit in Applied Microbiology and Pharmacology of natural substances of the University of Abomey-Calavi prior to use. During this period, the animals had free access to food and water. Animal Research Review Panel and Animal Welfare Unit regulation of temperature and lighting systems was maintained with a room temperature of 20–26°C as well as regular light cycles of 12 hours light/dark. All methods and protocols used in this study were observed following established public health guidelines “Guide for Care and Use of Laboratory Animals” [29].

### 2.3. Phytochemical Analysis

A qualitative phytochemical screening carried out according to the method based on precipitation and staining reactions described by Houghton and Raman [30] made it possible to detect the presence or absence of large chemical groups in the organs of the selected plants. These searched chemical groups were tannins, gallic tannins, flavonoids, anthocyanins, leucoanthocyanins, alkaloids, mucilages, reducing compounds, sterols, terpenes, and saponosides.

### 2.4. Extracts Production

Samples collected of the selected plants were cleaned with tap water and then dried at ambient temperature in the shade at the Research Unit in Applied Microbiology and Pharmacology of natural substances (URMAPha). After drying, they were then reduced to powder using a Retsch SM 2000/1430/Upm/Smf electric mill. From this powder, two types of extraction of each organ of the medicinal plants studied were carried out, following the methodology described by Klotoé et al. [31]. These were the aqueous extraction and the hydroethanolic extraction (50% water-ethanol; v/v). Thus, fifty (50) grams of powder were macerated in 500 mL of solvent (water and water-ethanol). The mixture was stirred continuously for 72 hours at room temperature. The homogenate obtained was filtered three times on absorbent cotton and once on Whatman No. 1 filter paper. The filtrate obtained was then evaporated at a temperature of 40°C in an oven (drying oven) until a dry mass was obtained which represents the extract. The extracts thus produced were placed in the refrigerator at 4°C and put back into solution during the various tests.

### 2.5. Larval Cytotoxicity Test

The cytotoxic effect of the powders of the studied plants was evaluated on the *Artemia salina* larvae. The method described by Dougnon et al. [32] was adopted in this study. *Artemia salina* larvae were obtained by hatching 10 mg of *Artemia salina* eggs placed under continuous agitation in 1 L of seawater for 72 hours. Dilution series of order 2 of a stock solution of plant powder with a concentration of 20 mg/mL were carried out in order to have an increasing scale of concentration. To 1 mL of each of these diluted solutions was added 1 mL of seawater containing 16 live larvae. A control solution without the extract was prepared under the same conditions. All solutions were incubated under agitation for 24 hours. Counting the number of dead larvae in each solution under an optical microscope produced a representative curve of the number of surviving larvae versus the concentration of the extract. The data (concentration-response) were log-transformed, and the LC_50_ (mean lethal concentrations) was determined. To assess the larval toxicity of the extract, the correlation grid associating the degree of toxicity to the LC_50_ proposed by Moshi et al. [33] was used. According to this grid, if the LC_50_ value is higher than 0.1 mg/mL, the extract is declared nontoxic. If this value is between 0.1 and 0.5 mg/mL, the extract is slightly toxic, and if the LC_50_ is less than 0.1 mg/mL, the extract is toxic.

### 2.6. Acute Toxicity Test

The rats were divided into lots according to their weight. Since the extracts were administered orally, the method described in OECD guideline 423, the acute toxicity class method, was adopted. Since the selected plant species are commonly used by the population and no major toxic effects were reported, a toxicity limit test with a single dose of 2000 mg/kg body weight was used. Twelve hours before the toxicity tests were conducted, the animals were deprived of food and water. After weighing the rats, 21 groups of three rats were assembled and treated (Table 2).

During the experiment, the animals were monitored and observed individually twice a day (morning and evening) over a period of 14 days. A data collection sheet was drawn up for each rat in order to collect possible signs of toxicity (skin and hair changes, the appearance of edema, walking backward, breathing difficulties, morbidity, and mortality). At the end of the treatment, the rats were deprived of food the last night before sampling. Blood samples (Day 0 and Day 14) were taken by puncture of the retroorbital sinus for all animals under ether anesthesia. The blood sample was collected in two types of tubes, one containing EDTA and the other without anticoagulant (dry tube). The samples from EDTA tubes were intended for hematological analysis. The dry tubes are centrifuged at 4000 rpm for 10 minutes, and the resulting serum is stored at −20°C for analysis of biochemical parameters. After sampling, two animals per lot were sacrificed under anesthesia with ether for the collection of organs such as the liver and kidney. These organs were rinsed with 0.9% saline and fixed in 10% buffered formalin.

#### 2.6.1. Hematological and Biochemical Examinations

These examinations were performed at the Research Unit in Applied Microbiology and Pharmacology of natural substances (URMAPha). The hematological examinations included red and white blood cell counts, hemoglobin level, hematocrit, Mean Globular Volume (MGV), Mean Corpuscular Hemoglobin Content (MCH), and determination of Mean Corpuscular Hemoglobin Concentration (MCHC). The biochemical tests were concerned with the determination of urea, creatinine, aspartate aminotransferase (ASAT), and alanine aminotransferase (ALAT).

#### 2.6.2. Histological Examinations

Histological sections of the liver and kidneys were performed at the histopathology Laboratory of the Institute of Applied Biomedical Sciences (ISBA) of the University of Abomey-Calavi. The pathomorphological study consisted of hematoxylin-eosin staining of thin sections of 5 *μ*m thicknesses. It is routine staining after which the nuclei, stained by hematoxylin, appear dark blue, and the cytoplasm, stained by eosin, appears pink. The microscopic observation of these sections was carried out with the ZEISS camera microscope at different magnifications so that only the most representative photographs were selected.

### 2.7. Data Analysis

The obtained data were subjected to statistical analysis using SPSS 26.0. Quantitative variables were presented as mean and standard deviation. Qualitative variables were presented in percentages. The probit analysis was used for the LC_50_ determination. The student test was used to compare the values of the different biochemical and hematological parameters of the treated animals with those of the control lot. The weight gain of the rats in each of the test lots was also compared to that of the control lot via Student's *t*-test. The significance threshold was set at 5%.

## 3. Results

The data obtained in this study are related to the phytochemical screening of the studied plants, larval cytotoxicity, and the different parameters of acute toxicity.

### 3.1. Qualitative Phytochemical Analysis of the Studied Plants

Table 3 presents the results of the phytochemical screening of samples of the plants studied. From this table, it should be noted that the plants' extracts present a varied richness in secondary metabolites. Thus, we note the presence of tannins in all the samples of the plants studied with a strong presence in the powder of *Vernonia amygdalina*, *Pterocarpus erinaceus*, and *Senna italica*. As for flavonoids, which belong to the group of phenolic compounds in the same way as tannins, their presence was reported in all plants studied except *Senna italica, Anacardium occidentale*, and *Manihot esculenta.* Moreover, except *Pterocarpus erinaceus*, saponosides are identified in all plants studied. The same observation is made concerning the reducing compounds which were identified in all plants except *Manihot esculenta*. Alkaloids were detected in five of the ten plants studied. These are *Khaya senegalensis*, *Ocimum gratissimum, Daniellia oliveri*, *Diospyros mespiliformis*, and *Vernonia amygdalina.*

#### 3.1.1. Larval Cytotoxicity

**Figure 1:**
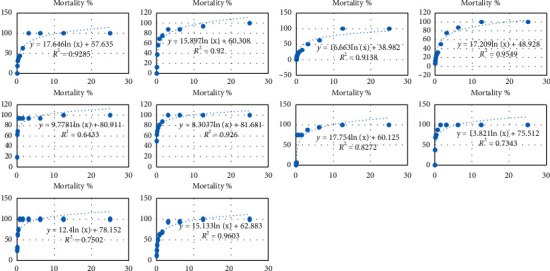
Sensitivity of *Artemia Salina* larvae to the studied medicinal plants.

The sensitivity of *Artemia Salina* larvae of the different extracts of the plants studied is presented in Figure 1. It emerges increased mortality of *Artemia Salina* larvae as the concentration of extracts of the plants studied increases. LC_50_ obtained for the studied medicinal plants ranges from 0.02 to 1.93 mg/mL (Table 4). By reporting LC_50_ values obtained at the scale established by Moshi et al. [33], it appears that, at the concentrations tested, plants with LC_50_ values between 0.10 and 1.93 mg/mL are noncytotoxic (LC_50_ ≥ 0.1 mg/mL). On the other hand, plants such as *Senna italica* and *Daniellia oliveri* have medium cytotoxicity (0.05 mg/mL > LC_50_ ≥ 0.1 mg/ml).

### 3.2. Acute Toxicity

#### 3.2.1. LD_50_ of the Studied Plant Extracts

No mortality was noted in the animals of the different lots at the doses tested (2000 mg/kg). Similarly, no signs of apparent toxicity were observed. The LD_50_ of the studied plants is thus higher than 2000 mg/kg.

#### 3.2.2. Weight Change of Animals in Different Lots

Data collected on the body weight of the rats during the experiment are summarized in Table 5. From this table, a weight growth of the Wistar rats should be noted in all the groups. This weight growth was significant for the treated rats with *Anacardium occidentale*, *Senna italica,* and *Manihot esculenta* aqueous extract (*p* < 0.05).

The influence of the different treatments on the biochemical parameters of treated and control rats is summarized in Table 6. From this table, it appears that the plant extracts studied had no significant influence on the different biochemical parameters compared to the control group (*p* > 0.05). However, there was a significant decrease in ASAT levels in rats treated with the aqueous extract of *Ocimum gratissimum* (*p* < 0.05).

#### 3.2.3. Effect of the Studied Plant Extracts on Hematological Parameters

Tables 7 and 8 present, respectively, the influence of the studied plants on the parameters of the erythrocytic and leukocytic lineages. Statistical analysis of the data presented in these tables shows that, compared to the Wistar rats in the control group, all the extracts of the ten plants studied have no significant effect on these different hematological parameters of the animals (*p* > 0.05).

#### 3.2.4. Histopathology Study

**Figure 2:**
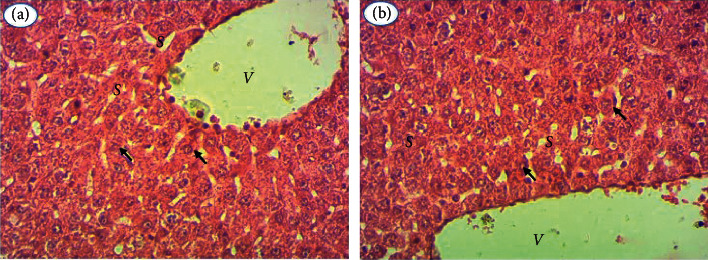
Hepatic histology of rats treated with extracts of the plants studied and control rats (A), 400x magnification.

**Figure 3:**
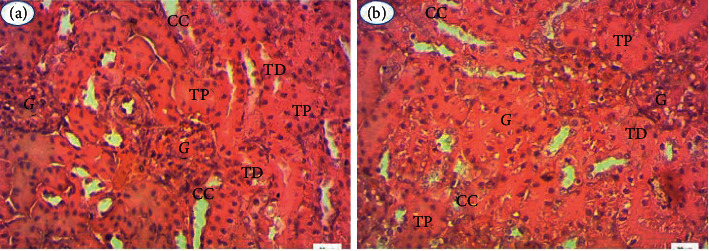
Kidney histology of rats treated with the plant extracts studied (B) and control rats (A), 400x magnification.

From kidney and liver tissues, histological sections were performed to confirm the hematological and biochemical data. For all extracts, histological sections of the organs of treated rats show no structural abnormalities compared to controls. Figures 2 and 3 show, respectively, the hepatic and renal histology of extract-treated and control rats.

The hepatic parenchyma of treated rats (B) has a typical appearance as observed in control rats (A). The hepatocytes (arrows) have a normal appearance and are arranged in cords separated by sinusoids (S). The sinusoids drain into the centrilobular vein (V).

The renal parenchyma of treated rats (B) has the typical architecture observed in control rats (A). The glomeruli (G), proximal tubes (TP), distal tubes (TD), and collecting channels (CC) are well identifiable. The extracts, therefore, did not affect the renal structures in any way.

## 4. Discussion

The present study aimed to explore the composition in secondary metabolites and reveal the toxicological characteristics of ten plants of the Beninese flora used in the traditional treatment of diarrheal diseases.

The qualitative phytochemical screening of the plants studied revealed a varied richness in bioactive molecules. The presence of catechic tannins, gallic tannins, flavonoids, anthocyanins, and sterol-terpenes, alkaloids, saponosides, and reducing compounds were detected. Among these bioactive compounds, tannins are identified in all the plants studied. Similar observations are reported in the literature [34–39]. This presence of tannins as well as those of other identified bioactive molecules evokes the medicinal properties of the plants studied and their therapeutic uses in several pharmacopoeias. Indeed, plants containing tannins have astringent, hemostatic, antiseptic, and toning properties [40]. In addition, the tannins contained in medicinal plants are endowed with antimicrobial [41, 42], antiviral, anti-inflammatory, antihypertensive, antimutagenic, immunostimulant, and antitumor properties [43–45]. Several reports have documented the efficacy of tannins in the treatment of diarrheal diseases [46–48]. As for flavonoids, it has been reported that plant extracts, which are rich in flavonoids, have antimicrobial activity [49, 50]. A study has shown that flavonoids associated with saponins isolated from *Anacardium occidentale,* one of the medicinal plants in this study, are useful in the treatment of diarrhea [51]. Alkaloids, one of the metabolites identified in this study, are nitrogenous organic substances with antibacterial and antifungal properties [52]. The above evidence supports the use of the plants studied in the treatment of many diseases including diarrheal diseases.

The safety of the plants was evaluated in vitro in the *Artemia Salina* model and by acute oral toxicity according to the OECD 423 protocol. The cytotoxic effect of the extracts evaluated according to the *Artemia Salina* model was used as a preliminary safety screening. The data obtained for this test indicate that, with the exception of *Senna italica* and *Daniellia oliveri,* all other plants studied are noncytotoxic to *Artemia Salina* larvae at the concentrations tested. Similar observations were reported by Dehou et al. [53] and Déguénon et al. [54] for *Ocimum gratissimum* and Soha et al. [55] for *Khaya senegalensis*. Lagarto Parra et al. [56] demonstrated a good correlation (*r* = 0.85; *p* < 0.05) between this larval toxicity test and toxicological effects on a whole animal. Some authors have confirmed this hypothesis in their work [57–59]. However, such an extrapolation is hotly debated [60]. In order to confirm the data of this preliminary screening, on the one hand, and to explore the safety of the studied plants on a whole organism, on the other hand, the acute oral toxicity test in Wistar albino rats was carried out. The results obtained indicate that no mortality was recorded during the 14 days of the experiment. Similarly, no signs of apparent toxicity were observed. The plants studied thus have an LD_50_ higher than 2000 mg/kg. In the literature, it is reported that plants with an LD_50_ greater than 1000 mg/kg orally are considered nontoxic [61]. In addition, during the experiment, the evolution of the body weight of the animals was monitored. The data obtained for this parameter indicate weight growth in all animals' lots, reflecting their good physiological condition. This information suggests that the aqueous and hydroethanolic extracts of the ten plants studied show no apparent toxicity at the dose of 2000 mg/kg. Similar observations have been reported in the literature by several authors for different extracts of the plants studied. Indeed, Konan et al. [37] and Jintanaporn et al. [62] reported that, at 2000 mg/kg, hydroethanolic extract from the leaves of *Anacardium occidentale* induced no mortality or signs of apparent toxicity. The same observation is reported by Ahmadu et al. [63] for the ethanolic extract of *Daniellia oliveri*. Ebbo et al. [64] demonstrated the safety of the methanolic extract of the leaves of *Diospyros mespiliformis* at a dose of 5000 mg/kg in Wistar rats. Nadro and Modibo [65] have also proven the safety of aqueous and ethanolic extracts of *Senna italica* in their work. As for *Pterocarpus erinaceus*, Ajayi et al. [34] showed that the ethanolic extract of the leaves of this plant induced no apparent toxicity in Wistar rats at a dose of 5000 mg/kg.

The study of Nkoua Badzi et al. [38] reached the same conclusion with regard to the aqueous extract of *Rauvolfia vomitoria.* Legba et al. [66] showed the safety of aqueous and ethanolic extracts of the leaves of *Vernonia amygdalina* used in Southern Benin. Ojo et al. [40] and Satish and Ranjana [67] reported the safety of ethanolic aqueous extracts of the leaves of *Manihot esculenta* and *Ocimum gratissimum*, respectively. For *Khaya senegalensis*, the data obtained from this study are consistent with those reported by Soha et al. [55].

Beyond the data on the apparent toxicity of medicinal plants, adverse effects can be observed at the tissue scale through functional and structural alterations of certain vital organs such as the liver and kidneys. These aspects were taken into account in the present study by exploring renal, hepatic, and hematological parameters as well as a histopathological study of these organs. The exploration of the hepatic function concerned the measurement of the enzymatic activity of transaminases (ASAT, ALAT). ALAT and ASAT are the main markers of liver function generally explored in toxicological studies of medicinal plants. Under normal physiological conditions, these markers are present at low concentrations in serum. Elevated serum levels of these enzymes, particularly ALAT, are considered sensitive markers of liver damage [68, 69]. In this study, no significant influence was noticed on the activity of these enzymes. However, it is reported that the nonsignificant increase in the levels of these enzymes observed in both the test and control lots is not related to any functional impairment of the liver but rather to stress due to manipulation. This hypothesis is reinforced by the histopathological study which revealed that no structural alterations of the liver were observed.

The renal parameters evaluated in the exploration of kidney function were uremia and creatininemia. Any increase in the level of these markers indicates probable kidney tissue damage [70]. In this study, it should be noted that no significant effect was induced by the treatments with the various plant extracts studied. These data, which reflect an absence of functional alteration of the kidneys, are reinforced by the histological study which revealed an absence of structural alteration of this organ.

Moreover, the hematopoietic system is one of the most sensitive targets for toxic substances [71]. It represents an important marker of the physiological and pathological state of humans and animals. Any alteration in hematological parameters is perceived as a potential risk of anemia [72]. Data from the present study indicate that the plants studied had no significant effect on either erythrocyte or leukocyte lineage parameters. This suggests that the extracts of the ten plants studied were not responsible for any anemic disorders. However, the increase in the number of leukocyte lineage cells (white blood cells and monocytes) observed could be explained by the fact that there was a possible thrombocytosis coupled with hyperleukocytosis. This could be attributed to the use of the metallic gastric tube. Dougnon et al. [73] support this idea by reporting that since the test lots were force-fed and the control batch had free access to water, it is possible that the probe may have attacked the esophageal wall of these animals causing an acute syndrome.

From the abovementioned data, it appears that the different extracts of the studied plants were not responsible for any mortality and any alteration of biochemical and hematological parameters. They do not present any risk of toxicity when used at a dose of 2000 mg/kg.

## 5. Conclusion

The purpose of this study was to generate recent data on the phytochemical and toxicological characteristics of ten (10) plants used in the traditional treatment of diarrheal diseases. Phytochemical screening of the plants studied revealed a varied richness of secondary metabolites. Two approaches were adopted for safety testing: acute oral toxicity and larval cytotoxicity. The results obtained following the larval cytotoxicity model indicated that the plants studied are noncytotoxic. Acute toxicity data indicate that the plants studied did not induce mortality or structural and functional alterations of the liver and kidneys at the dose of 2000 mg/kg. These observations justify the use of these plants in several African pharmacopeias. However, more in-depth toxicological studies (subacute, chronic, and subchronic toxicity) are necessary for a better knowledge of the toxicological profile of these plants.

## Figures and Tables

**Table 1:** Information about in the selected medicinal plants.

Identification number	Scientific name	Botanical family	Used part	Collection area (municipality)	Collection period
YH 434/HNB	*Anacardium occidentale* L.	Anacardiaceae	Leaves	Abomey-Calavi	July 2020
YH 436/HNB	*Daniellia oliveri* (Rolfe) Hutch. & Dalziel	Leguminosae	Leaves	Toffo	July 2020
YH 438/HNB	*Diospyros mespiliformis* Hochst*. ex A. DC.*	Ebenaceae	Leaves	Toffo	July 2020
YH 435/HNB	*Khaya senegalensis* (Desr.) A. Juss.	Meliaceae	Bark	Abomey-Calavi	July 2020
YH 442/HNB	*Manihot esculenta* Crantz	Euphorbiaceae	Leaves	Abomey-Calavi	July 2020
YH 437/HNB	*Ocimum gratissimum* L.	Lamiaceae	Leaves	Abomey-Calavi	July 2020
YH 440/HNB	*Pterocarpus erinaceus* Poir.	Euphorbiaceae	Leaves	Toffo	July 2020
YH 441/HNB	*Rauvolfia vomitoria* Afzel.	Apocynaceae	Leaves	Porto-novo	August 2020
YH 432/HNB	*Senna italica* Mill.	Leguminosae	Leaves	Tanguieta	July 2020
YH 439/HNB	*Vernonia amygdalina* Delile	Asteraceae	Leaves	Abomey-Calavi	July 2020

**Table 2:** Treatment of constituted animal lots.

Lots	Lot type	Substances administered	Medicinal plants
1	Control	Distilled water	
2	Test	Aqueous extract	*Anacardium occidentale*
3	Test	Hydroethanolic extract	*Anacardium occidentale*
4	Test	Aqueous extract	*Daniellia oliveri*
5	Test	Hydroethanolic extract	*Daniellia oliveri*
6	Test	Aqueous extract	*Diospyros mespiliformis*
7	Test	Hydroethanolic extract	*Diospyros mespiliformis*
8	Test	Aqueous extract	*Manihot esculenta*
9	Test	Hydroethanolic extract	*Manihot esculenta*
10	Test	Aqueous extract	*Ocimum gratissimum*
11	Test	Hydroethanolic extract	*Ocimum gratissimum*
12	Test	Aqueous extract	*Khaya senegalensis*
13	Test	Hydroethanolic extract	*Khaya senegalensis*
14	Test	Aqueous extract	*Pterocarpus erinaceus*
15	Test	Hydroethanolic extract	*Pterocarpus erinaceus*
16	Test	Aqueous extract	*Rauvolfia vomitoria*
17	Test	Hydroethanolic extract	*Rauvolfia vomitoria*
18	Test	Aqueous extract	*Senna italica*
19	Test	Hydroethanolic extract	*Senna italica*
20	Test	Aqueous extract	*Vernonia amygdalina*
21	Test	Hydroethanolic extract	*Vernonia amygdalina*

**Table 3:** Qualitative phytochemical screening of the studied medicinal plants.

Secondary metabolites	Test reagents	AO	DM	DO	KS	Me	OG	PE	RV	SI	VA
Tannins	Ferric chloride	+	+	+	+	+	+	++	+	++	++
Catechic tannins	Stiasny's reagent	+	+	+	+	+	+	++	−	++	−
Gallic tannins	Ferric chloride and saturation with sodium acetate	+	+	−	−	+	+	++	−	−	++
Flavonoids	Shinoda test with powder magnesium	−	+	+	+	−	+	++	+	−	++
Anthocyanins	Hydrochloric acid and ammonia at 50%	−	−	−	−	−	+	−	−	−	++
Leuco-anthocyanins	Hydrochloric acid	+	+	+	+	−	−	−	−	−	−
Alkaloids	Mayer's reagent	−	+	+	+	−	+	−		−	++
Mucilage	Absolute alcohol test	−	−	+	+	−	−	−	+	++	++
Reducing compounds	Test with fehling liqueur	+	+	+	+	−	+	+	+	+	+
Sterol-terpenes	Anhydride acetic-sulfuric acid	−	−	−	−	−	−	++	+	−	++
Saponosides	Foam index test	+	+	+	+	+	+	−	+	+	+

+: presence; -: absence; ++: Strong presence. AO: Anacardium occidentale; DM: Diospyros mespiliformis; DO: Daniellia oliveri; KS: Khaya senegalensis; ME: Manihot esculenta; OG: Ocimum gratissimum; PE: Pterocarpus erinaceus; RV: Rauvolfia vomitoria; SI: Senna italica; VA: Vernonia amygdalina.

**Table 4:** LC_50_ of the studied medicinal plants and their interpretation.

*Plants*	LC_50_ (mg/mL)	R^2^
*Anacardium occidentale*	0.65	0.93
*Daniellia oliveri*	0.04	0.64
*Diospyros mespiliformis*	1.93	0.91
*Khaya senegalensis*	1.06	0.95
*Manihot esculenta*	0.52	0.92
*Ocimum gratissimum*	0.1	0.75
*Pterocarpus erinaceus*	0.15	0.73
*Rauvolfia vomitoria*	0.56	0.83
*Senna italica*	0.02	0.92
*Vernonia amygdalina*	0.43	0.96

**Table 5:** Evolution of the body weight of the Wistar rats.

Parameters	Rat weight				
Plants	Extracts	Day 0	Day 14	Gain/loss	Gain/loss (%)
Control	D. W	137 ± 5.29	155.66 ± 4.16	18.67	13.62
*Anacardium occidentale*	H_2_0	131.33 ± 5.13	160.33 ± 6.66	29	22.08 ^*∗*^
	EtH_2_0	140.33 ± 3.51	124.67 ± 5.03	4.33	3.09
*Daniellia oliveri*	H_2_0	132.33 ± 4.73	146.67 ± 5.51	14.33	10.83
	EtH_2_0	139.67 ± 8.08	140.67 ± 2.52	1	0.716
*Diospyros mespiliformis*	H_2_0	134.67 ± 6.43	136.33 ± 3.78	1.67	1.24
	EtH_2_0	157 ± 2	155.67 ± 8.50	5.33	3.39
*Khaya senegalensis*	H_2_0	147 ± 6	134.33 ± 0.58	4	2.72
	EtH_2_0	135 ± 3	136 ± 4	1	0.74
*Manihot esculenta*	H_2_0	134.33 ± 3.79	167 ± 8.18	32.67	24.32 ^*∗*^
	EtH_2_0	162.67 ± 11.93	172.67 ± 4.16	10	6.15
*Ocimum gratissimum*	H_2_0	138 ± 7.55	124.67 ± 10.69	3.33	2.41
	EtH_2_0	134 ± 3.46	134.33 ± 6.03	0.33	0.25
*Pterocarpus erinaceus*	H_2_0	138.67 ± 5.13	149 ± 7	10.33	7.45
	EtH_2_0	137.33 ± 3.05	141.33 ± 5.03	4	2.91
*Rauvolfia vomitoria*	H_2_0	138 ± 2.64	154 ± 9.16	16	11.59
	EtH_2_0	160 ± 3.46	132.67 ± 1.53	6	3.75
*Senna italica*	H_2_0	135.33 ± 2.51	163 ± 3.61	27.67	20.44 ^*∗*^
	EtH_2_0	131.67 ± 4.72	147.67 ± 6.81	16	12.15
*Vernonia amygdalina*	H_2_O	130 ± 7.94	140.33 ± 4.73	10.33	7.95
	EtH_2_0	132 ± 7.93	131 ± 6.56	1.33	1.01

H_2_O: aqueous extract; EtH_2_O: hydroethanolic extract; DW: distilled water.  ^*∗*^ Significant difference with the control group (*p* < 0.05). 3.2.3. Effect of the Studied Plant Extracts on the Biochemical Parameters of Rats

**Table 6:** Effects of plant extracts on biochemical parameters in Wistar rats.

Parameters		Urea (g/L)		Creatinine (mg/L)		ASAT (UI/L)		ALAT (UI/L)	
Plants	Lots/Extracts	Day 0	Day 14	Day 0	Day 14	Day 0	Day 14	Day 0	Day 14
*Control*	D. W	0.79 ± 0.08	0.63 ± 0.04	7.89 ± 1.14	12.51 ± 0.93	255.33 ± 20.03	334.33 ± 23.28	86.78 ± 5.44	142.57 ± 35.07
*A. occidentale*	EtH_2_0	0.79 ± 0.05	0.84 ± 0.09	8.21 ± 0.34	13.29 ± 1.46	254 ± 16.37	321.67 ± 21.08	88.93 ± 0.85	129.59 ± 40.92
	H_2_0	0.86 ± 0.07	0.61 ± 0.04	8.33 ± 0.68	9.78 ± 4.03	252.67 ± 9.02	303.33 ± 50.74	103.83 ± 18.75	154.03 ± 15.49
*D. mespiliformis*	EtH_2_0	0.74 ± 0.07	0.74 ± 0.11	7.48 ± 1.18	12.36 ± 0.69	272.67 ± 12.70	342.67 ± 2.89	83.07 ± 3.77	104.52 ± 13.45
	H_2_0	0.77 ± 0.17	0.77 ± 0.06	7.99 ± 0.71	9.67 ± 2.60	262.33 ± 31.78	248 ± 28.35	81.07 ± 17.45	151.12 ± 10.16
*D. oliveri*	EtH_2_0	0.92 ± 0.11	0.70 ± 0.15	6.88 ± 2.02	10.14 ± 0.91	237.33 ± 28.31	258.67 ± 56.36	105.29 ± 14.22	145.73 ± 9.25
	H_2_0	0.73 ± 0.04	0.64 ± 0.03	8.22 ± 0.10	8.93 ± 1.59	266 ± 38.93	230.67 ± 48.33	84.96 ± 2.87	142.91 ± 14.57
*K. senegalensis*	EtH_2_0	0.84 ± 0.06	0.67 ± 0.10	7.77 ± 0.28	6.91 ± 2.57	252.33 ± 7.63	297.33 ± 9.07	98.26 ± 19.91	123.90 ± 34.04
	H_2_0	0.83 ± 0.07	0.75 ± 0.08	7.61 ± 0.61	11.11 ± 1.02	293.33 ± 13.31	258 ± 72.13	96.82 ± 19.22	77.54 ± 6.37
*M. esculenta*	EtH_2_0	0.75 ± 0.08	0.64 ± 0.04	7.54 ± 0.65	10.09 ± 2.43	251.67 ± 23.71	354.67 ± 42.33	105.76 ± 21.77	149.49 ± 5.72
	H_2_0	0.73 ± 0.03	0.57 ± 0.02	7.15 ± 0.20	10.45 ± 2.91	255 ± 19.97	361.33 ± 34.53	90.2 ± 5.88	130.41 ± 13.92
*O. gratissimum*	EtH_2_0	0.65 ± 0.02	0.64 ± 0.06	5.94 ± 0.49	10.06 ± 0.92	247.33 ± 14.74	299 ± 40.71	96.37 ± 13.02	120.99 ± 16.38
	H_2_0	0.88 ± 0.10	0.70 ± 0.07	7.93 ± 0.30	13.02 ± 1.17	301.33 ± 8.08	238.67 ± 23.46 ^*∗*^	97.26 ± 9.70	116.97 ± 14.31
*P. erinaceus*	EtH_2_0	0.77 ± 0.06	0.62 ± 0.14	6.61 ± 1.24	8.73 ± 2.22	290.67 ± 58.04	288.33 ± 30.09	85.09 ± 3.13	112.55 ± 17.31
	H_2_0	0.77 ± 0.03	0.93 ± 0.08	7.14 ± 1.36	11.23 ± 0.20	256 ± 29.86	273.67 ± 11.60	78.24 ± 23.37	114.18 ± 20.03
*R. vomitoria*	EtH_2_0	0.72 ± 0.20	0.75 ± 0.13	7.83 ± 0.34	10.84 ± 0.57	256 ± 24	318.33 ± 19.53	86.9 ± 6.75	189.32 ± 39.20
	H_2_0	0.55 ± 0.02	0.71 ± 0.10	6.81 ± 0.71	10.33 ± 2.46	278 ± 38	325.33 ± 49.54	95.35 ± 10.21	163.45 ± 16.50
*S. italica*	EtH_2_0	0.73 ± 0.02	0.48 ± 0.12	6.19 ± 0.96	10.37 ± 0.24	226.33 ± 14.57	271.33 ± 28.58	88.53 ± 3.97	113.48 ± 21.30
	H_2_0	0.67 ± 0.05	0.53 ± 0.10	6.87 ± 0.09	8.36 ± 0.79	320.67 ± 27.68	374.33 ± 63.35	84.11 ± 16.36	111.77 ± 25.90
*V. amygdalina*	EtH_2_0	0.72 ± 0.01	0.76 ± 0.09	8.05 ± 0.70	12.40 ± 0.51	257.33 ± 19.21	288.33 ± 30.09	84.19 ± 18.67	103.60 ± 14.26
	H_2_0	0.8 ± 0.08	0.59 ± 0.12	7.767 ± 0.62	10.17 ± 1.33	266 ± 22.54	324.67 ± 33.56	95.58 ± 4.88	130.30 ± 11.55

H_2_0: aqueous extract; EtH_2_0: hydroethanolic extract; DW: distilled water.

**Table 7:** Effect of the studied plant extracts on the erythrocyte lineage parameters of the Wistar rat.

Parameters		RB (T/L)		Hte (%)		Hb (g/dl)		MGV (fL)		MCH (Pg)		MCHC (g/dl)	
Plants	Lots	Day 0	Day 14	Day 0	Day 14	Day 0	Day 14	Day 0	Day 14	Day 0	Day 14	Day 0	Day 14
Control	D. W	6.54 ± 1.14	6.67 ± 0.41	41.33 ± 7.28	37.8 ± 3.83	13.86 ± 2.86	14.36 ± 0.86	63.2 ± 2.23	56.56 ± 2.23	32.2 ± 20.35	21.5 ± 0.53	33.43 ± 1.04	38.06 ± 1.66
*D. oliveri*	EtH_2_0	6.62 ± 0.46	6.49 ± 0.42	38.36 ± 2.56	36.83 ± 2.63	13.13 ± 1.26	13.86 ± 1.11	58 ± 20.01	56.7 ± 0.95	19.8 ± 1.05	21.33 ± 0.38	34.13 ± 1.00	37.66 ± 0.81
	H_2_0	6.36 ± 0.38	6.58 ± 0.41	44.26 ± 2.04	37.3 ± 1.99	14.16 ± 0.74	13.9 ± 0.9	69.86 ± 7.46	59.53 ± 0.93	22.3 ± 0.46	21.4 ± 1.08	32.13 ± 3.18	37.33 ± 1.30
*A. occidentale*	EtH_2_0	6.21 ± 1.18	7.48 ± 1.60	40.06 ± 6.63	44.36 ± 10.51	13.26 ± 2.05	16.66 ± 3.41	64.76 ± 2.40	59.2 ± 1.63	21.5 ± 1.1	22.33 ± 0.58	33.16 ± 0.45	37.73 ± 1.09
	H_2_0	7.16 ± 0.79	5.54 ± 0.90	44.4 ± 4.79	32.33 ± 5.25	14.56 ± 1.26	12.06 ± 1.35	61.93 ± 2.78	58.3 ± 0.61	20.36 ± 0.83	21.86 ± 1.41	32.9 ± 0.79	37.46 ± 1.76
*D. mespiliformis*	EtH_2_0	6.28 ± 0.34	6.65 ± 0.26	43.56 ± 3.85	37.83 ± 0.98	13 ± 0.43	14.5 ± 0.78	69.33 ± 5.19	57 ± 1.99	20.76 ± 1.65	21.8 ± 1.08	30.03 ± 2.75	38.23 ± 1.06
	H_2_0	6.24 ± 0.41	6.62 ± 0.38	39.9 ± 3.38	36.5 ± 1.80	12.73 ± 1.10	13.9 ± 0.74	63.9 ± 1.45	55.06 ± 0.47	20.46 ± 1.10	21 ± 0.1	32 ± 1.49	38.16 ± 0.21
*K. senegalensis*	EtH_2_0	6.1 ± 0.56	7.34 ± 1.30	37.23 ± 2.77	40.10 ± 8.28	13.23 ± 1.02	14.37 ± 2.54	61.03 ± 4.51	54.40 ± 2.86	21.67 ± 0.25	19.53 ± 1.01	35.57 ± 2.22	35.90 ± 1.06
	H_2_0	5.52 ± 0.63	7.71 ± 0.77	35.30 ± 5.03	42.43 ± 2.52	11.87 ± 2.11	15.47 ± 0.81	63.39 ± 4.47	57.70 ± 3.82	21.5 ± 2.23	21.93 ± 2.31	35.56 ± 1.20	36.73 ± 040
*M. esculenta*	EtH_2_0	6.12 ± 0.79	4.48 ± 3.05	37.43 ± 2.14	24.4 ± 16.32	12.77 ± 1.11	9.56 ± 6.49	61.77 ± 7.12	55.37 ± 2.45	21.00 ± 1.31	21.50 ± 0.79	34.13 ± 1.82	38.87 ± 1.15
	H_2_0	6.12 ± 1.12	5.34 ± 0.46	43.9 ± 3.61	33.20 ± 3.20	13.3 ± 2; 15	11.83 ± 1.30	72.87 ± 8.52	62.10 ± 4.17	21.87 ± 1.50	22.13 ± 0.91	30.13 ± 2.42	35.63 ± 1.70
*O. gratissimum*	EtH_2_0	4.81 ± 0.73	5.71 ± 1.36	29.83 ± 7.41	32.33 ± 8.05	10.47 ± 2.32	12.06 ± 3.27	65.9 ± 3.80	56.53 ± 1.36	22.36 ± 0.76	21.00 ± 1.30	33.67 ± 2.20	37.2 ± 1.38
	H_2_0	5.92 ± 0.32	6.83 ± 0.24	39.33 ± 1.55	39.56 ± 2.50	12.73 ± 0.71	14.33 ± 0.65	65.10 ± 1.32	57.87 ± 2.51	20.93 ± 0.76	20.93 ± 0.21	33.43 ± 0.59	36.27 ± 1.68
*P. erinaceus*	EtH_2_0	6.15 ± 0.26	6.67 ± 0.35	39.93 ± 0.90	38.13 ± 3.14	13.06 ± 0.55	14.36 ± 0.81	65.03 ± 3.92	57.13 ± 2.01	21.3 ± 0.36	21.56 ± 0.64	32.76 ± 1.70	37.76 ± 2.29
	H_2_0	5.45 ± 1.47	6.78 ± 0.42	35.86 ± 10.17	37.46 ± 1.11	11.33 ± 2.62	13.5 ± 0.26	65.63 ± 2.34	55.36 ± 1.77	20.96 ± 0.98	19.93 ± 1.11	32 ± 2.25	35.7 ± 0.62
*R. vomitoria*	EtH_2_0	5.90 ± 0.12	6.66 ± 0.14	38.73 ± 0.60	39.9 ± 1.56	13.03 ± 0.25	14.13 ± 0.66	65.6 ± 1.47	57.33 ± 2.72	22.1 ± 0.5	21.16 ± 0.60	33.76 ± 0.11	37.3 ± 0.17
	H_2_0	6.61 ± 1.07	5.73 ± 1.22	40.1 ± 3.04	32.76 ± 6.86	13.8 ± 0.2	11.93 ± 2.20	66.73 ± 2.54	57.2 ± 0.86	17.86 ± 8.60	20.9 ± 0.95	26.5 ± 12.15	36.53 ± 1.26
*S. italica*	EtH_2_0	6.17 ± 0.42	6.22 ± 0.71	40.46 ± 2.10	35.7 ± 1.75	13.4 ± 0.46	13.4 ± 0.36	65.6 ± 2.15	57.73 ± 5.31	21.73 ± 0.94	21.66 ± 2.35	33.1 ± 1.65	37.5 ± 0.89
	H_2_O	6.00 ± 1.13	7.01 ± 0.83	37.1 ± 6.97	28.93 ± 6.38	12.2 ± 2.38	14.4 ± 0.96	61.83 ± 0.21	58.33 ± 0.58	20.23 ± 0.21	21.16 ± 0.30	32.73 ± 0.29	36.66 ± 0.38
*V. amygdalina*	EtH_2_0	6.48 ± 0.47	6.28 ± 0.26	40.1 ± 1.68	36.76 ± 1.00	13.3 ± 1.04	14.06 ± 0.68	62.03 ± 1.81	58.56 ± 0.98	20.5 ± 0.46	22.4 ± 0.53	33.06 ± 1.26	38.26 ± 1.29
	H_2_0	6.46 ± 0.26	6.25 ± 0.31	38.76 ± 1.72	37.2 ± 3.25	14.1 ± 0.1	14.63 ± 0.71	59.9 ± 1.12	59.33 ± 2.22	21.83 ± 0.84	23.4 ± 0.1	36.5 ± 1.65	39.43 ± 1.59

H_2_0: aqueous extract; EtH_2_0: hydroethanolic extract; DW: distilled water. RB: red blood cells; Hb: hemoglobin; Hte: hematocrit; MGV: Mean Globular Volume; MCH: Mean Corpuscular Hemoglobin. MCHC: Mean Corpuscular Hemoglobin Concentration.

**Table 8:** Effect of the studied plant extracts on the leukocyte lineage parameters of the Wistar rat.

Parameters		White blood cell (G/L)		Neutrophilic Polynuclear (%)		Monocytes (%)		Lymphocytes (%)	
Plants	Lots	Day 0	Day 14	Day 0	Day 14	Day 0	Day 14	Day 0	Day 14
Control	D. W	5.06 ± 0.76	6.9 ± 0.7	6.76 ± 3.02	23.96 ± 8.17	6.03 ± 3.86	11.03 ± 2.33	87.2 ± 0.85	65 ± 11.00
*D. oliveri*	EtH_2_0	7.73 ± 1.19	7.3 ± 1.67	12.83 ± 1.75	21.56 ± 0.11	4.7 ± 2.53	10.13 ± 2.80	82.46 ± 1.15	68.3 ± 2.88
	H_2_0	4.23 ± 1.25	6.83 ± 0.81	6.8 ± 3.27	26.06 ± 2.04	7 ± 1.45	10 ± 0.70	86.2 ± 4.43	64 ± 2.15
*A. occidentale*	EtH_2_0	5.2 ± 1.9	5.73 ± 1.81	6.16 ± 2.83	26.03 ± 3.28	6.46 ± 1.33	11.73 ± 1.85	87.36 ± 1.50	62.23 ± 4.99
	H_2_0	7.96 ± 2.58	10.43 ± 5.48	17.26 ± 7.10	29.1 ± 17.84	8.06 ± 4.36	9.96 ± 1.84	74.67 ± 9.25	60.93 ± 19.39
*D. mespiliformis*	EtH_2_0	7.13 ± 1.83	9.5 ± 3.18	20.76 ± 1.65	21.8 ± 1.08	30.03 ± 2.75	38.23 ± 1.07	77.23 ± 17.15	57.63 ± 3.52
	H_2_0	5.66 ± 2.42	10.3 ± 3.34	4.8 ± 1.21	30.1 ± 1.9	6.63 ± 0.38	12.33 ± 0.84	88.56 ± 1.59	57.56 ± 2.60
*K. senegalensis*	EtH_2_0	5.63 ± 2.48	6.06 ± 3.44	5.00 ± 2.77	27.87 ± 8.40	4.30 ± 1.61	14.30 ± 1.25	90.70 ± 1.25	57.83 ± 7.54
	H_2_0	7.17 ± 1.47	9.53 ± 2.14	7.33 ± 0.68	27.03 ± 7.42	5.73 ± 1.44	13.1 ± 0.87	86.93 ± 1.10	59.86 ± 7.18
*M. esculenta*	EtH_2_0	6.13 ± 0.32	4.97 ± 3.09	16.63 ± 1.90	22.6 ± 8.27	2.47 ± 2.67	11.47 ± 1.36	80.9 ± 2.10	65.93 ± 9.00
	H_2_0	4.07 ± 0.41	9.63 ± 1.08	8.00 ± 3.38	26.83 ± 7.65	6.87 ± 2.48	9.17 ± 4.68	85.13 ± 4.80	64.00 ± 12.31
*O. gratissimum*	EtH_2_0	6.17 ± 0.64	7.33 ± 0.87	13.83 ± 1.86	22.23 ± 2.70	6.17 ± 1.20	11.13 ± 0.46	80.00 ± 1.12	66.63 ± 2.73
	H_2_0	9.37 ± 1.46	4.93 ± 1.59	4.83 ± 0.31	26.53 ± 5.80	5.56 ± 0.61	12.4 ± 1.21	89.6 ± 0.34	61.07 ± 5.46
*P. erinaceus*	EtH_2_0	6.73 ± 2.04	7.93 ± 4.13	6.43 ± 2.72	23.03 ± 3.40	5.3 ± 1.25	12.86 ± 0.83	88.26 ± 2.15	64.1 ± 3.96
	H_2_0	4.96 ± 0.76	10.43 ± 3.69	3.83 ± 2.22	18.76 ± 9.12	4.93 ± 2.75	10.1 ± 4.09	91.23 ± 2.15	71.13 ± 13.2
*R. vomitoria*	EtH_2_0	6.56 ± 0.55	8.86 ± 1.87	9.66 ± 10.53	27.43 ± 2.38	10.06 ± 6.71	10.83 ± 3.08	80.26 ± 17.24	61.73 ± 5.03
	H_2_0	5.8 ± 0.1	7.53 ± 2.60	11.84.75	29.56 ± 13.47	9.1 ± 8.07	9.6 ± 0.95	79.1 ± 12.40	60.83 ± 14.25
*S. italica*	EtH_2_0	7.03 ± 2.01	8.16 ± 2.45	7.46 ± 6.05	17.5 ± 1.13	4.66 ± 0.60	10.23 ± 1.00	87.86 ± 5.63	72.26 ± 2.00
	H_2_0	9.83 ± 2.59	12.96 ± 1.55	6.77 ± 2.06	15.23 ± 4.54	6.76 ± 1.32	7.73 ± 2.32	86.46 ± 3.12	74.7 ± 2.42
*V. amygdalina*	EtH_2_0	5.5 ± 1.9	9.8 ± 2.16	7.03 ± 3.69	24.7 ± 5.30	5.43 ± 1.28	12.66 ± 2.11	87.53 ± 4.70	62.63 ± 6.74
	H_2_0	6.23 ± 2.61	6.2 ± 1.91	10.26 ± 5.48	21.36 ± 2.45	5.53 ± 1.19	11.4 ± 2.6	84.2 ± 6.07	67.23 ± 4.30

H_2_O: aqueous extract; EtH_2_O: hydroethanolic extract; DW: distilled water.

## Data Availability

The data used to support the findings of this study are fully available upon request from the corresponding author.
